# Essential Oil Composition and Micromorphological Traits of *Satureja montana* L., *S. subspicata* Bartel ex Vis., and *S. kitaibelii* Wierzb. Ex Heuff. Plant Organs

**DOI:** 10.3390/plants10030511

**Published:** 2021-03-09

**Authors:** Tanja Dodoš, Smiljana Janković, Petar D. Marin, Nemanja Rajčević

**Affiliations:** Faculty of Biology, University of Belgrade, 11000 Belgrade, Serbia; smiljana.jankovic@bio.bg.ac.rs (S.J.); pdmarin@bio.bg.ac.rs (P.D.M.); nemanja@bio.bg.ac.rs (N.R.)

**Keywords:** essential oils, *Satureja montana*, *Satureja subspicata*, *Satureja kitaibelii*, micromorphological traits, plant organs, chemophenetics

## Abstract

The essential oil (EO) composition of *Satureja* plants is highly variable. Recent studies suggest that there is an even difference in the EO composition from different plant organs within the same plant. This study aims to examine the chemical profile of EOs and the micromorphological characteristics of different organs of three *Satureja* species. The relationship between the number of glandular trichomes and EOs profile and relative yield is also investigated. Individuals from five populations were visualized using a scanning electron microscope, while EOs of leaves, calyces, corollas, and whole aerial parts were isolated using simultaneous distillation and extraction and analyzed using gas chromatography/mass spectrometry. Three types of glandular trichomes were detected. Peltate trichomes were present on all plant organs of studied species, while two types of capitate trichomes show different organ and species preferences. The EOs profiles differed across the plant parts, but showed a species specific composition. Univariate and multivariate statistics were used to show a correlation between the peltate trichomes and EO yield, and chemophenetic significance of EO profiles.

## 1. Introduction

*Satureja* L. genus (Lamiaceae) has around 30 species of annual and perennial shrubs and semi-shrubs distributed in arid areas on sunny and open rocky cliffs [1]. *S. montana* L. is a widely distributed species, while *S. subspicata* Vis. and *S. kitaibelii* Wierzb. Ex Heuff. are both endemic [1]. *S. subspicata* is endemic to the Dinaric Alps (Italy, Slovenia, Croatia, Bosnia and Herzegovina, Montenegro, and Albania), and the *S. kitaibelii* is endemic to the Balkan Mountains (southwest Romania, eastern Serbia, and northwest Bulgaria) [1,2]. These species are used as aromatic and medicinal herbs since they contain substantial quantities of essential oils, particularly *S. montana* [3]. All studied species are used as herbs and teas in traditional medicine for the treatment of different illnesses, e.g., respiratory system ailments (bronchitis or cough), and lymphatic nodule inflammation, and for improving overall blood health [4]. *S. montana* and *S. kitaibelii* are also used for urinary, digestive disorders, and as an aphrodisiac [5,6,7].

The essential oils (EOs) are complex mixtures of volatile components with intensive odor and various colors [8,9]. These components have different biological functions for plants such as the attraction of pollinators or protection against herbivores or microorganisms [8,10,11]. EOs are stored in specialized structures such as secretory glands, cavities, channels, and glandular trichomes. In the Lamiaceae family, EOs are stored in glandular trichomes. These trichomes are found on the surface of stems, leaves, calyces, and corollas of plants from the *Satureja* genus [12,13,14,15]. They are roughly divided into two groups—peltate and capitate glandular trichomes. Peltate glandular trichomes are composed of one basal epidermal cell, one wide stalk cell, and a multicellular head consisting of 8, 12 or 16 cells. Two types of capitate trichomes are described, type 1 (C1) composed of one basal epidermal cell, one stalk cell, and unicellular secretory head, and type 2 (C2) which are the smallest and have one basal cell, one or two stalk cells, and a unicellular head [12,13,15,16]. Peltate glandular trichomes are also found on all organs, while capitate trichomes show organ preference [12]. Previous research showed that *S. montana* has more peltate trichomes on the outer side of calyx than *S. subspicata* [12], but the distribution of trichomes varies between species and organs. Non-glandular trichomes are found on all organs, *S. montana* has a denser indumentum compared to the other two species. *S. subspicata* and *S. kitaibelii* have fewer non-glandular trichomes on the leaves and calyces, while on the stem, these trichomes are present only on two opposite sides [1,12,13,14].

Previous investigations of the biological activity of EOs of all three species show antimicrobial [17,18,19,20,21,22,23,24] and antioxidative activity [25,26]. The aforementioned activities largely depend on the dominant component in the essential oil profile, which differs between the studied species. In *S. kitaibelii*, the EO profile is dominated by monoterpenes *p*-cymene, geraniol, limonene or linalool [27,28]. In *S. subspicata,* dominant compounds were sesquiterpenes *α*-eudesmol, spathulenol, *β*-eudesmol, (*E*)-caryophyllene and monoterpenes *α*-pinene, carvacrol, and thymol [12,17,22,25,28]. The *S. montana* profile is also dominated by monoterpenes, carvacrol, *p*-cymene, thymol, geraniol, myrcene or linalool [21,28,29,30,31]. The essential oil profiles also show a significant difference between taxa. In essential oils of *S. subspicata* and *S. kitaibelii,* several components have relatively high percentages (12–30%), rarely one component is above 50%, while *S. montana* essential oils usually have one dominant component (20–64%).

The chemical composition of EOs is quite variable, as it is influenced by the drying method [32], distillation method [33,34], and the plant itself due to the different compositions of EO between plant organs [35,36]. These differences are closely related to the organ function, e.g., basil (*Ocimumbasilicum*) and lilac sage (*Salvia verticillata*) leaf essential oils have insect repellent properties, while flower EOs have pollinator attraction properties [37,38]. Research shows that the highest yield of EO is in a period of flowering [39,40,41,42], which could be explained by the contribution of essential oils from flowers (calyces and corollas). Moreover, several authors have found a correlation between the number of peltate trichomes and essential oil yield [43,44]. Moreover, peltate and capitate glandular trichomes have a different chemical composition, the peltate oil profile is much more complex, while capitate is simpler [36]. Interestingly, the chemical composition of oil in peltate glands varies in quantities of individual components even between other peltate glands on the same organ, e.g., leaf [36,45]. Although the essential oils composition has been studied in all three species from several aspects, the literature survey shows that only the essential oils profile from *S. kitaibelii* plant organs have been investigated [27].

**Figure 1:**
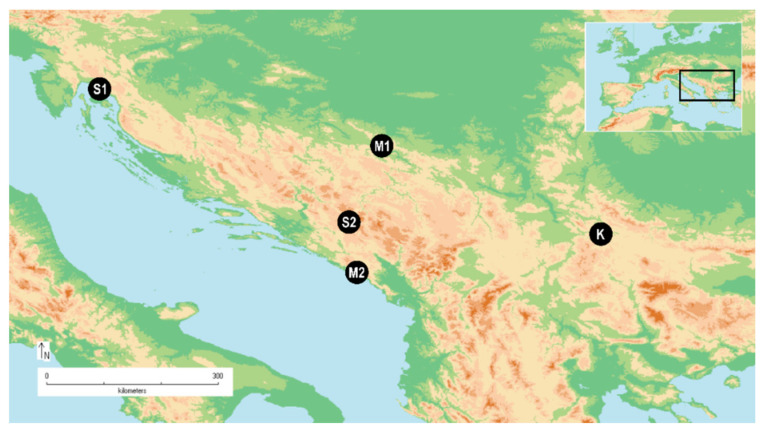
Spatial distribution of studied samples S1: *Satureja subspicata* subsp. *liburnica* (Croatia); S2: *S. subspicata* subsp. *subspicata* (Montenegro); M1: *S. montana* (Montenegro) coastal; M2: *S. montana* (Bosna and Herzegovina) continental; K: *S. kitaibelii* (Serbia).

This research was carried out to determine the composition of the EO and micromorphological features of different plant organs of three *Satureja* species which have different distributions, and their possible relation. The micromorphological investigation of *S. kitaibelii* is done for the first time. The studied plants were chosen based on the different geographic distribution patterns. Two taxa with a narrow distribution, *S. kitaibelii* and *S. subspicata* (both subspecies included), and one widely distributed, *S. montana*. Two samples of *S. montana* were selected based on the literature data that suggest different chemotypes in individuals from mountain and coastal regions [40,46] (Figure 1). Additionally, the relationship between the number and type of glandular trichomes, and essential oil profile and relative yield, has been investigated.

## 2. Results

### 2.1. Microscopic Analysis

All studied samples had both non-glandular and glandular trichomes. Non-glandular trichomes were unicellular to multicellular, unbranched, and uniseriate. All three species had non-glandular trichomes on stems, calyces, and corollas. Species differed in the number and trichome length, as well as in their distribution (Table 1).

**Figure 2:**
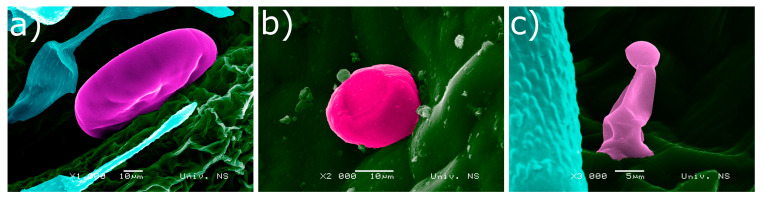
Scanning electron micrographs of glandular trichomes found in analyzed taxa. (**a**) Peltate glandular trichome; (**b**) type 1 capitate glandular trichome (C1); (**c**) type 2 capitate glandular trichome (C2); SEM micrographs colored using Portable Photoshop CS6.

**Figure 3:**
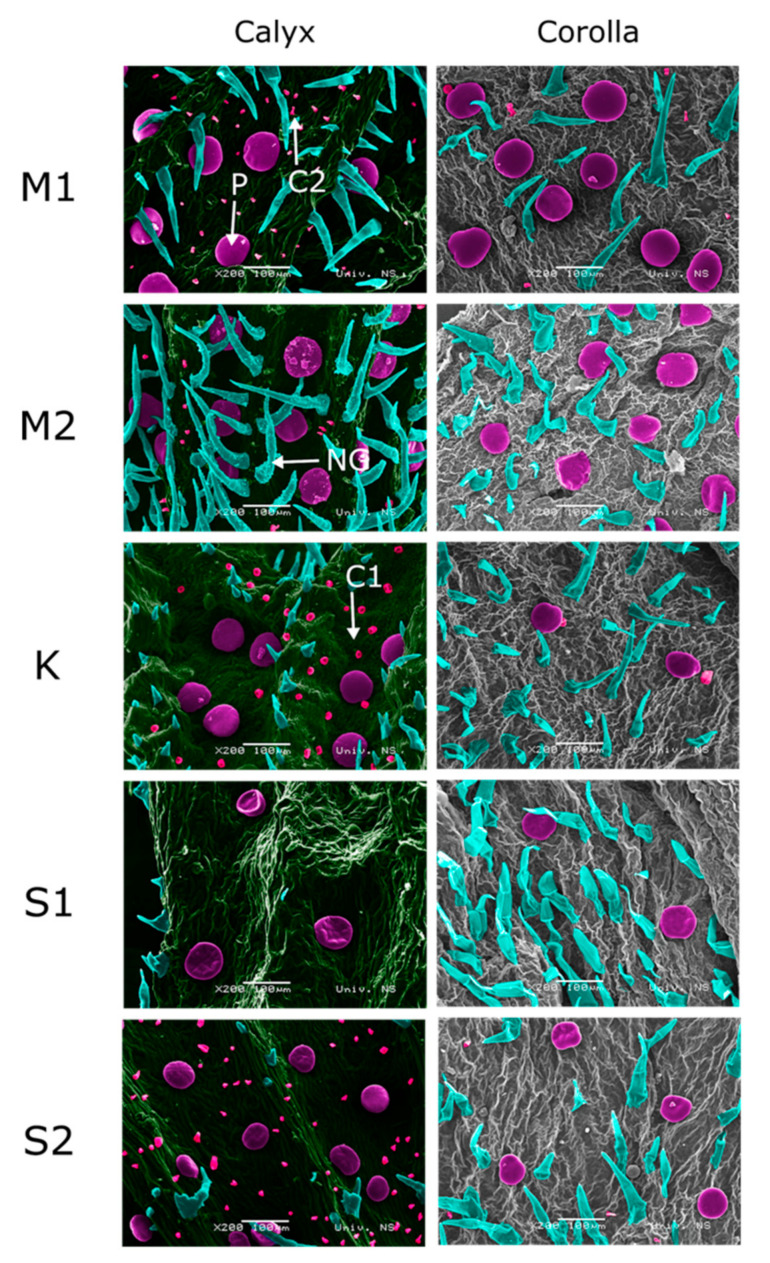
Scanning electron micrographs of M1: *S. montana* continental; M2: *S. montana* coastal; K: *S. kitaibelii*; S1: *S. subspicata* subsp. *liburnica*; S2: *S. subspicata* subsp. *subspicata* calyces and corollas. P: Peltate trichomes; C1: Type 1 capitate trichomes; C2: Type 2 capitate trichomes; NG: Non-glandular trichome; SEM micrographs colored using Portable Photoshop CS6.

**Figure 4:**
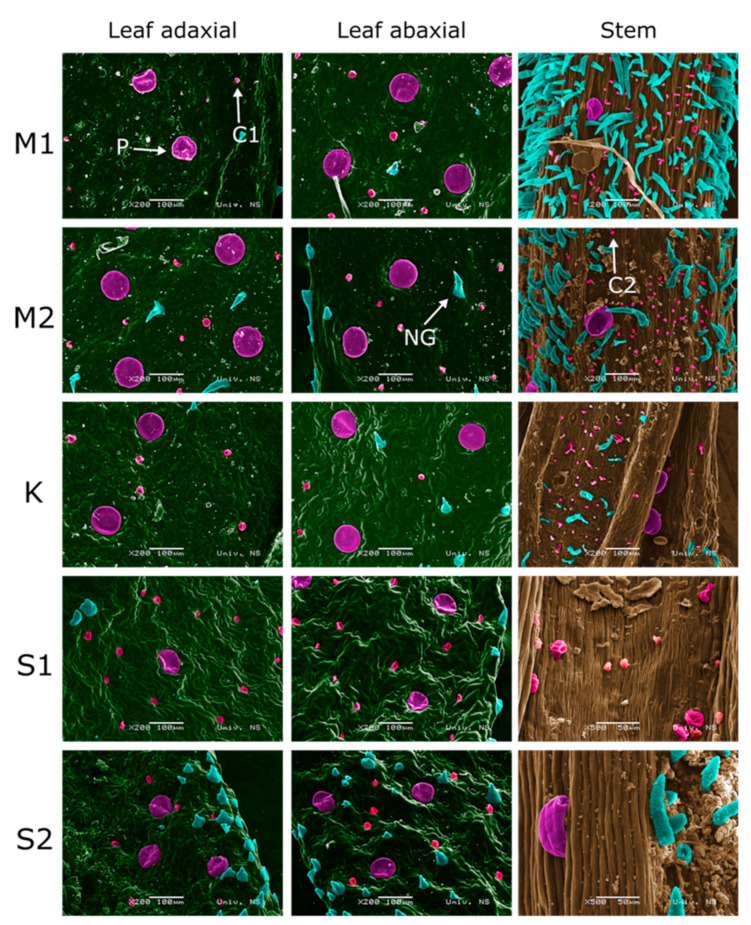
Scanning electron micrographs of M1: *S. montana* continental; M2: *S. montana* coastal; K: *S. kitaibelii*; S1: *S. subspicata* subsp. *liburnica*; S2: *S. subspicata* subsp. *subspicata*, leaves and stems. P: Peltate trichomes; C1: Type 1 capitate trichomes; C2: Type 2 capitate trichomes; NG: Non-glandular trichome; SEM micrographs colored using Portable Photoshop CS6.

SEM micrographs revealed three types of glandular trichomes: Peltate, capitate type 1 (C1), and type 2 (C2) (Figure 2). Peltate trichomes were present in all studied species and all plant parts, the only difference between the samples was in the number of these trichomes on a particular plant part. In most samples, peltate trichomes were more numerous in the flower region (slightly more numerous on calyces) and less numerous on both sides of leaves and stems (Table 1, Figure 3 and Figure 4). The size of these trichomes was an average of 74 ± 9 μm in all studied samples.

Unlike peltate trichomes, capitate C1 and C2 show organ preference. Capitate trichomes (C1) resemble peltate trichomes, with basal and neck cells sunken into the epidermis, but are three times smaller (20 ± 4 μm). This type of glandular trichomes was present on the leaves in all samples, being more numerous on the abaxial leaf side (Figure 4). These trichomes were also present on all parts of *S. kitaibelii*, on stems of *S. subspicata* subsp. *liburnica*, and on calyces of *S. subspicata* subsp. *subspicata* (Table 1, Figure 3 and Figure 4). Type 2 capitate trichomes (C2) are the smallest ones (6 ± 2 μm), and were found on stems of *S. montana*, *S. kitaibelii*, and *S. subspicata* subsp. *Liburnica*, as well as on calyces of *S. montana* and *S. subspicata* subsp. *subspicata* (Figure 3 and Figure 4).

### 2.2. Essential Oil Yield and Composition

Fourteen to 60 compounds were detected in the EO composition, accounting for 90.4 to 99.8% of the total oil (Table S2). The EO profiles differed greatly between the studied taxa and used plant part (Table 2). When comparing EOs obtained from the aboveground plant material (herba), *Satureja montana* and *S. kitaibelii* EOs were strongly dominated by monoterpenes, while EOs of *S. subspicata* subspecies had higher abundances of sesquiterpenes.

**Figure 5:**
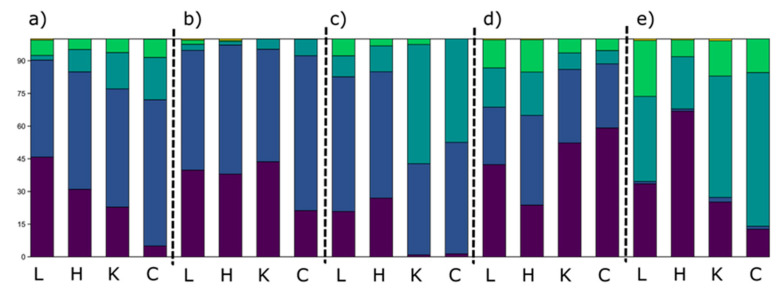
Percentages of different terpene classes in studied *Satureja* EOs from leaves, herba, calyx, and corolla. (**a**) *S. montana* subsp. *montana* from the mountain region; (**b**) *S. montana* subsp. *montana* from the coastal region; (**c**) *S. kitaibelii*; (**d**) *S. subspicata* subsp. *liburnica*; (**e**) *S. subspicata* subsp. *subspicata*; 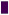: Monoterpene hydrocarbons; 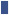: Oxygenated monoterpenes; 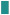: Sesquiterpene hydrocarbons; 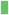: Oxygenated sesquiterpenes; 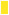: Other aliphatic and aromatic hydrocarbons; L: Leaf; H: Herba; K: Calyx; C: Corolla.

The essential oil profiles of studied taxa showed distinctive patterns. In *S. montana* from the continental population (M1), between 44 and 58 compounds were detected in EOs. There were distinctive quantitative differences between EOs from different plant parts (Figure 5). The EOs in leaves were dominated by monoterpenes, with almost equal parts of monoterpene hydrocarbons and oxygenated monoterpenes, while sesquiterpenes and other compounds represented only a small percentage of the total oil. The EO from herba showed a somewhat intermediate composition, positioning itself closer to leaf EO. The leaf EO was dominated by *p*-cymene, while corolla and calyx EOs had a significantly higher percentage of sesquiterpenes, with geraniol as a dominant component (Table 3).

The same holds true for the second individual sampled from the coastal area (M2), but here the domination of the monoterpenes was even more pronounced. In this sample, *p*-cymene was the dominant component in leaves, and thymol the second most abundant. In all other samples, thymol was the dominant, while the second most abundant compound was γ-terpinene. Even though thymol had a high abundance in all EOs, flower EOs had a higher percentage of thymol (39.4–51.7% vs. 25.7–32.0%). The EO profile of this chemotype was much simpler, with only 23 to 34 detected components. The EO from herba had an intermediate composition, with its composition more similar to the flower EOs, especially calyx EO.

In *S. kitaibelii,* the EO composition of individual plant parts and organs differs not only in the profile but also in its complexity. The EOs from flower parts contained fewer compounds (14 and 20 in corolla and calyx, respectively) in comparison to leaves and herba (60 and 54, respectively). The profiles also differed significantly. The EO from leaves was strongly dominated by monoterpenes, while EOs from flower parts were dominated by oxygenated monoterpenes and sesquiterpenes. The herba EO was more similar to leaf EO than flower EOs. Even though linalool was the dominant component in all samples, *p*-cymene was abundant in EOs from leaves and herba, while (*E*)-caryophyllene and germacrene D were abundant in EOs from flower parts.

The EO profiles of two *Satureja* subspicata subspecies also differed. *S. subspicata* subsp. *liburnica* had EOs strongly dominated by monoterpenes, while EOs of *S. subspicata* subsp. *subspicata* were dominated by sesquiterpenes. The differences between plant parts were also present in these two taxa. In *S. subspicata* subsp. *liburnica* (S1) calyx and corolla, EOs monoterpenes were much more abundant than in leaf EO, while in *S. subspicata* subsp. *subspicata* (S2), the EO of leaves had a higher abundance of oxygenated sesquiterpenes. In both taxa, in all samples, α-pinene was the most abundant compound, however, the second most abundant constituents differed. In subsp. *liburnica*, borneol dominated in leaf and herba EOs, while camphene and camphor were more abundant in flower parts. In subsp. *subspicata*, spathulenol was more abundant in leaf EO, while germacrene D was the second most abundant in flower parts. The herba EO of subsp. *subspicata* was strongly dominated by α-pinene, and the second most abundant compound was germacrene D, though only 6.1%.

### 2.3. Statistical Analysis

**Figure 6:**
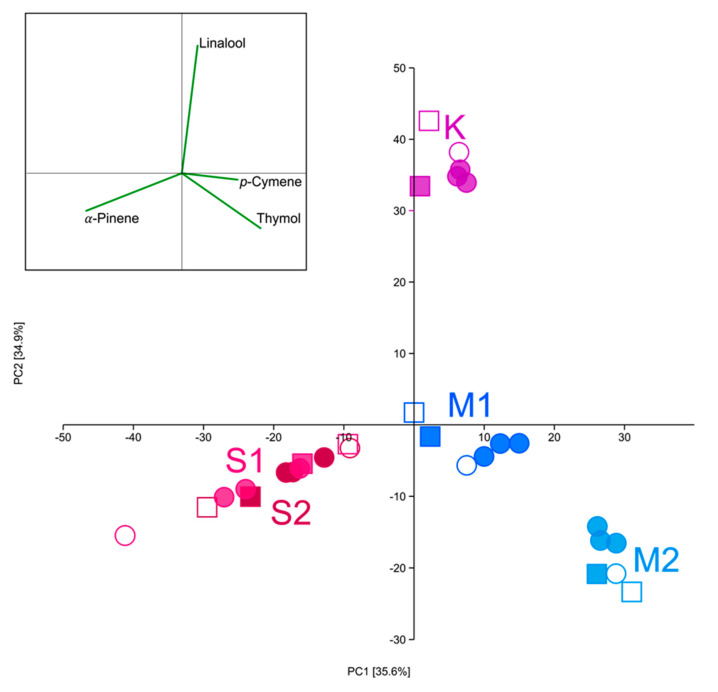
The principle component analysis (PCA) scatter plot with all samples based on 16 components from the EO with principle vectors; M1: *Satureja montana* from the mountain region; M2: *Satureja montana* from the coastal region; K: *S. kitaibelii*; S1: *S. subspicata* subsp. *liburnica;* S2: *S. subspicata* subsp. *subspicata*; ■: Calyx; □: Corolla; •: Leaf; ◯: Herba.

Sixteen components of EO, that were present on average above 0.5%, and did not show a correlation were chosen for multivariate statistical analysis. Even though dominant components and EO profiles differed within the plant between different parts (i.e., leaves, calyces, and corollas), all analyzed taxa separated on the PCA scatter plot (Figure 6). The first two eigenvectors explained 70.5% of the total variability. Both subspecies of *Satureja subspicata* are separated from *S. montana* and *S. kitaibelii* on the first axis. Two samples of *S. montana*, one continental and one coastal, further separated on both PC axes. *S. kitaibelii* further separated from *S. montana* on the second axis. However, two subspecies of *S. subspicata* also separated on the second axis, though only slightly in comparison to the other samples. This is mainly due to a simpler EO profile, with α-pinene being the dominant compound in both taxa. Four EO components were responsible for the separation of all taxa: α-pinene for the separation of *S. subspicata*, thymol and *p*-cymene for the separation of *S. montana*, and linalool for the separation of *S. kitaibelii*.

To test the correlation between the micromorphological features and essential oil yield and composition, correlation tests were performed. Spearman’s correlation test showed a moderate correlation between the corrected FID area and the number of peltate trichomes (r*_s_*= 0.60 *p* = 0.02), while there was no significant correlation between the corrected FID area and the number of any other type of glandular trichomes or a total number of glandular trichomes, suggesting that peltate trichomes attribute most to the essential oil yield. When the total volume of glandular trichomes was calculated, Spearman’s correlation test showed a slight increase in the correlation coefficient (r*_s_* = 0.61 *p* = 0.02). Due to the very different chemical composition of EOs between species, there was no correlation found between the EO profile and glandular trichome type or number.

## 3. Discussion

Previous investigations of leaf micromorphology in *S. montana* and *S. subspicata* showed the presence of peltate glandular and variance in the presence of capitate glandular trichomes. The type 1 capitate trichomes (C1) found here were described previously [12,13,14]. Later research also found two more types of capitate trichomes that were not observed in our samples. Capitate trichomes (C1) in the studied samples show different distribution patterns between all three species, suggesting that these trichomes could be an additional taxonomic parameter for separation at the species level. However, a further study involving a greater number of samples from the entire distribution range is necessary. Type 2 capitate trichomes (C2) were previously found in *S. horvatii* [47] (although in that study trichomes were named digitiform on SEM micrographs) but were not reported earlier for the studied species. C2 trichomes varied mostly in the distribution patterns between all taxa, even including the difference between two populations of the same species from different localities (*S. montana*), which could be attributed to the high genetic variability present in this Mediterranean species. The present research showed that there are no differences between the studied *Satureja* species based on the total number of glandular trichomes, but there were significant differences between plant organs based on the number and type of glandular trichomes. This could be explained by different distributions of glandular trichomes that were uneven on plant organs.

The essential oil profiles of whole aerial parts (herba) for the studied species correspond to the literature in general. Dominant components in oil profiles of studied *S. montana* (M2) [12,17,21,28,29,31,48], *S. kitaibelii* [27], and *S. subspicata* (S1) [12,49,50] are in concordance with the literature data. However, germacrene D and *cis*-sabinene hydrate are reported for the first time as dominant components in the essential oil profile of *S. subspicata* (S2) and *S. montana* (M1), respectively. The EO profiles of individual organs, on the other hand, show different dominant components, which may or may not correspond to the herba EO profile. In *S. montana* (M2) and *S. subspicata* (S2), dominant components in the flower region EOs are also dominant in the herba EO, while in *S. subspicata* (S1), the dominant component in leaves is dominant in herba EO. In *S. kitaibelii,* linalool was dominant in the obtained EOs. On the other hand, the dominant component found in the herba EO of *S. montana* (M1) is the second most abundant compound in all organs.

Dominant components found in our EO profile, such as linalool, thymol, *p*-cymene, geraniol, α-pinene, and spathulenol, all exhibit antimicrobial activity [17,18,19,20,21,51]. Sabinene hydrate is used as a flavoring compound [52] and linalool, germacrene D, and thymol have insecticidal and fungicidal effects [38,53]. Geraniol, additionally, has been proven to reduce levels of angiotensin-converting enzyme 2 (ACE2) receptor, a host cell receptor with a crucial role in virus SARS-CoV-2 cell entry [54], and showed anti-inflammatory activity for CP-induced hepatotoxicity in rats [55]. The available literature shows higher antimicrobial activity of the whole EO rather than a single dominant component, due to the contribution of components in minor quantities which increase the activity [22]. However, a few studies found that even a single component (linalool) has a strong antimicrobial activity, due to the chemical structure (long-chain alcohol) which has higher water solubility [22].

Variability in the EO composition could be due to the density and EO profile of glandular trichomes among plants and organs. The present research showed a correlation between the number of peltate glandular trichomes and volume of glandular trichomes with the EO yield. In *S. kitaibelii,* differences between the EO profiles of plant organs were previously observed [27], and, additionally, in *S. hortensis*, quantitative variations between the EO profiles from peltate glands on different plant organs were found [56]. The difference in the quantities of EO components was present between peltate and capitate glands, on the same organ in *Salvia sclarea* [36], while in *Origanum vulgare* subsp. *hirtum*, differences in the chemical composition of EO were found also among peltate glands of the same organ [45]. This variation could be explained by the different biochemical capacities and distribution of the two gland types and by slight developmental differences of the oil glands [36]. Nonetheless, all these differences could contribute to the variability between the EO profiles of plant parts and herba. *S. subspicata* (S2) has the highest variation of leaves EO profile, dominant components in the three replicants varied from 19.6 to 35.6% (α-pinene) and 8.4–14.0% (spathulenol), while S1 had a somewhat lower variability for α-pinene (15.6–24.4%). Additionally, the herba EO sometimes possessed much higher percentages of dominant compounds than the individual leaves or flower parts (*S. kitaibelii*). In some of the herba EOs, dominant compounds were found in much lower abundances (*S. subspicata* S1) or were not detected at all (*S. montana* M1). A possible explanation is that due to a low and varying number of different glands (especially peltate) having different EO compositions on the leaves and stem could potentially give EOs a somewhat different composition when small samples are taken from the same individual. Further investigations are needed in order to assess the variability of individual glandular trichome EO composition in the studied species.

Even though the EO profile varied between plant parts, the EO profile of the analyzed species differed, showing that the chemical composition of essential oils is species specific. These findings further corroborate the use of essential oils in chemophenetic research. All these results show the important role of EO composition particularly when choosing plants for cultivation or from natural populations. *S. montana* and *S. kitaibelii* are used as food flavoring agents or herbal teas or tinctures, either from cultivation fields or from nature. In light of our results, an increment of the desired EO component could be achieved by harvesting a particular part or the whole plant, e.g., for the highest yield of thymol or geraniol flower region from *S. montana*, M2 and M1, respectively should be used, while for linalool whole aerial parts of *S. kitaibelii* could be used.

## 4. Materials and Methods

### 4.1. Plant Material

The plant material was collected from five populations (localities), two samples in Bosnia and Herzegovina (2016 and 2018), and one sample per the following countries: Croatia (2012), Montenegro (2018), and Serbia (2018). Each plant was in full bloom. The collected material was packed in paper bags and air-dried at room temperature in the shade for 7 days. Voucher specimens were deposited at the Herbarium of the University of Belgrade, Faculty of Biology (BEOU). Geographic details and voucher numbers of sampling localities are given in Table 4.

### 4.2. Micromorphological Analysis

Randomly chosen dried leaves, calyces, corollas, and stems were selected from each individual plant. They were coated with a thin layer of gold (ion sputtering coating) in the BAL-TEC SCD 005 Sputtering Device and observations were carried out on a JEOL JSM-6460LV electron microscope at 20 kV SEM. All micrographs were taken with a calibration scale at magnification 200 × 100 μm and then all trichomes were counted using the Digimizer software 4.3.5 (2005–2015). SEM micrographs were colored using Portable Photoshop CS6.

### 4.3. Essential Oils Isolation

For essential oils isolation, a single individual from each taxon was used, and from each of those plants dried leaves, calyces, and corollas (ca. 0.5–1.0 g), and whole aerial parts (grounded, ca. 2 g) were separately submitted to a 2 h simultaneous distillation and extraction (SDE) in the Likens-Nickerson-type apparatus [57]. The EO of aerial parts (herba) and flower parts were isolated once, while the EOs of leaves were done in triplicate to account for isolation procedure variability. Essential oils were dissolved in dichloromethane (CH_2_Cl_2_) and stored in amber glass vials at 4 °C until GC-FID and GC/MS analysis.

### 4.4. GC-FID and GC/MS Analysis

Settings and analysis procedures for GC-FID and GC/MS are as described in [58]. In all samples, the relative amounts of volatile components were expressed as percentages of the peak area of total ion chromatograms. Values under 0.05% were not considered during compound identification.

The relative essential oils yield of different plant organs were calculated as described in [27] for each sample individually. The split ratio varied from 25:1 to 3:1 depending on the essential oil yield. The injection volume (1 μL) was the same in all the samples.

### 4.5. Statistical Analyses

Data were analyzed using PAST 4.05 [59]. For all the samples, corrected FID areas were calculated based on the following formula: (Total area × split ratio)/dry weight.

For leaf EOs, the standard deviation and variance were calculated to assess the variability produced by the isolation procedure. In all statistical analyses, the mean values of leaf EOs and abundance of each terpene class were used. The principal component analysis (PCA) and Spearman’s linear correlation analysis were performed to assess the variability and relationship between data. For the PCA analysis, only components that were present on average above 0.5% and uncorrelated were used.

## Figures and Tables

**Table 1:** Number of non-glandular and glandular trichomes per 0.3 mm^2^ of *Satureja montana*, *S. kitaibelii*, and *S. subspicata* based on SEM micrographs.

Taxon	Code	Plant Part ^1^	NG	P	C1	C2
*Saturejamontana* subsp. *montana*	M1	Calyx	34	8	-	33
		Corolla	15	8	-	5
		Leaf adaxial	2	2	5	5
		Leaf abaxial	2	4	7	-
		Stem	182	1	-	28
	M2	Calyx	66	12	-	13
		Corolla	32	8	-	1
		Leaf adaxial	4	4	9	-
		Leaf abaxial	4	4	9	-
		Stem	76	2	-	47
*S. kitaibelii*	K	Calyx	48	8	29	-
		Corolla	39	2	2	-
		Leaf adaxial	1	2	5	-
		Leaf abaxial	5	3	4	-
		Stem	18	2	13	22
*S. subspicata* subsp. *liburnica*	S1	Calyx	8	3	2	-
		Corolla	38	2	-	-
		Leaf adaxial	-	1	12	-
		Leaf abaxial	4	3	13	-
		Stem	-	-	10	18
*S. subspicata* subsp. *subspicata*	S2	Calyx	15	10	4	55
		Corolla	23	4	-	4
		Leaf adaxial	26	4	6	-
		Leaf abaxial	25	3	8	-
		Stem	18	2	10	-

^1^ Material used for SEM micrographs; NG: Non-glandular trichomes; P: Peltate trichomes; C1: Type 1 capitate trichomes; C2: Type 2 capitate trichomes; M1: Continental region; M2: Coastal region.

**Table 2:** Essential oil profiles of *Satureja montana*, *S. kitaibelii*, and *S. subspicata*.

Taxon	EO ^1^	Yield ^2^	MH ^3^	MO ^4^	SH ^5^	SO ^6^	Other ^7^
M1	Leaf	1	44.34	43.11	2.09	6.85	0.46
	Calyx	4	21.82	56.29	15.92	5.97	-
	Corolla	3	4.72	63.9	18.52	7.94	2.54
	Herba	3	30.65	53.56	10.29	4.95	0.49
M2	Leaf	1	39.03	53.91	2.73	1.89	0.51
	Calyx	10	43.54	51.53	4.68	0.05	-
	Corolla	8	21.15	70.86	7.53	0.15	-
	Herba	6	26.13	56.39	1.58	0.5	0.85
K	Leaf	3	19.67	58.49	9.11	7.33	-
	Calyx	6	0.86	40.13	52.44	2.42	4.15
	Corolla	5	1.29	48.85	45.29	0	3.39
	Herba	4	26.13	56.39	11.54	3.07	0.85
S1	Leaf	1	40.85	25.48	17.4	12.39	0.42
	Calyx	1	49.91	32.84	7.2	6.13	-
	Corolla	1	58.78	29.27	6.03	5.31	-
	Herba	1	22.39	39.42	18.74	13.95	0.48
S2	Leaf	2	32.09	1.07	37.39	24.62	0.63
	Calyx	7	22.69	2.63	50.55	14.64	1.72
	Corolla	1	11.06	1.24	62.56	13.66	0
	Herba	1	65.76	1.01	23.59	7.56	2.15

^1^ Material used for EO isolation, ^2^ relative yields calculated as the ratio of total corrected FID integration area within each studied taxon separately; ^3^ MH: Monoterpene hydrocarbons; ^4^ MO: Oxygenated monoterpenes; ^5^ SH: Sesquiterpene hydrocarbons, ^6^ SO: Oxygenated sesquiterpenes; ^7^ other compounds including diterpenes, aliphatic, and aromatic non-terpenoid hydrocarbons; S1: *S. subspicata* subsp. *liburnica*; S2: *S. subspicata* subsp. *subspicata*; M1: *S. montanacontinental*; M2: *S. montana* coastal; K: *S. kitaibelii*.

**Table 3:** Three most abundant compounds in the plant part and herba essential oils (EOs) of *Satureja montana*, *S. kitaibelii*, and *S. subspicata*. The list of all compounds in their elution order is given in Table S2.

	Leaf	Calyx	Corolla	Herba
M1	*p*-Cymene (33.5%) *cis*-Sabinene hydrate (13.0%) Terpinen-4-ol (8.9%)	Geraniol (19.0%) *cis*-Sabinene hydrate (16.5%) *p*-Cymene (6.8%)	Geraniol (30.4%) *cis*-Sabinene hydrate (13.1%) *β*-Bourbonene (5.9%)	*cis*-Sabinene hydrate (24.8%) Germacrene D (3.1%) Bicyclogermacrene (2.7%)
M2	*p*-Cymene (30.0%) Thymol (29.6%) Thymoquinone (11.0%)	Thymol (39.4%) γ-Terpinene (28.4%) *cis*-Sabinene hydrate (5.0%)	Thymol (51.7%) γ-Terpinene (12.1%) *cis*-Sabinene hydrate (9.5%)	Thymol (40.5%) γ-Terpinene (18.8%) *p*-Cymene (11.9%)
K	Linalool (43.5%) *p*-Cymene (13.1%) Caryophyllene oxide (3.4%)	Linalool (38.3%) (*E*)-Caryophyllene (17.1%) Germacrene D (16.0%)	Linalool (48.3%) (*E*)-Caryophyllene (21.3%) Germacrene D (15.9%)	Linalool (47.3%) *p*-Cymene (13.2%) Limonene (4.6%)
S1	*α*-Pinene (20.8%) Borneol (7.3%) Limonene (7.0%)	*α*-Pinene (31.4%) Camphor (7.6%) Camphene (7.5%)	*α*-Pinene (40.3%) Camphene (10.2%) Camphor (7.9%)	*α*-Pinene (10.2%) Borneol (10.0%) Camphor (6.6%)
S2	*α*-Pinene (28.8%) Spathulenol (10.4%) Germacrene D (4.8%)	*α*-Pinene (18.9%) Germacrene D (16.5%) Spathulenol (8.7%)	Germacrene D (29.7%) *α*-Pinene (10.5%) (*E*)-Caryophyllene (9.5%)	Germacrene D (5.8%) Spathulenol (4.5%) (*E*)-Caryophyllene (2.9%)

M1: *S. montana continental*; M2: *S. montana* coastal; K: *S. kitaibelii*; S1: *S. subspicata* subsp. *liburnica*; S2: *S. subspicata* subsp. *subspicata*.

**Table 4:** Geographic and geologic characteristics of sampling sites for *S. montana*, *S. subspicata,* and *S. kitaibelii*.

Sample	Locality	Longitude [°]	Latitude [°]	Alt [m a.s.l.]	Voucher
M1	Zvornik, Bosnia and Herzegovina	44.3625	19.1117	186	17716
M2	Luštica, Montenegro	42.3943	18.7010	13	17717
K	Poganovo, Serbia	42.9811	22.6401	514	17460
S1	Jadranovo, Croatia	45.2365	14.6196	147	17232
S2	Gacko, Bosnia and Herzegovina	43.1679	18.5635	1132	17463

## Supplementary Materials

The following are available online at https://www.mdpi.com/2223-7747/10/3/511/s1. Table S1: Glandular trichomes of analyzed *Satureja* species; Table S2: Composition of plant and herba essential oil obtained from *Satureja montana*, *S. kitaibelii*, and *S. subspicata*.
