# Supplementary material for: Essential Oil Composition and Micromorphological Traits of Satureja montana L., S. subspicata Bartel ex Vis., and S. kitaibelii Wierzb. Ex Heuff. Plant Organs
Source: Plants (Basel). 2021 Mar 9;10(3):511. doi: 10.3390/plants10030511 (PMC8000019; doi:10.3390/plants10030511)
Supplement: Supplementary file 1 [file plants-10-00511-s001.zip › Supplemental_S1.pdf]

Table S1. Glandular trichomes of analized Satureja species

		**M1**[1]	**M2**	**K**	**S1**	**S2**
Calyx	P	79±5	77±5	71±7	75±12	75±12
	C1	-	-	16±2	23±7	18±3
	C2	6±1	7±1	-	-	5±1
Corolla	P	82±8	78±10	63±5	75±5	65±2
	C1	-	-	21±3	-	-
	C2	7±3.0	8	-	-	7±1
Leaf adaxial side	P	72±1	87±1	82±7	76	72±5
	C1	13±2	18±3	24±1	19±4	19±4
	C2	4±1	-	-	-	-
Leaf abaxial side	P	85±2	85±3	78±2	60±4	67±7
	C1	22±1	19±1	20±3	24±2	21±2
	C2	-	-	-	-	-
Stem	P	69	75±12	70±4	-	71.8
	C1	-	-	22±5	22±2	24.2
	C2	6±1	6±1	6±1	5±1	-

[1] – values in μm as mean ± SD where N>1; M1 – *Satureja montana* from mountain region; M2 – *S. montana* from coastal region; K – *S. kitaibelii;* S1 – *S. subspicata* subps. *liburnica;* S2 – *S. subspicata* subsp. s*ubspicata;* P – peltate trichomes; C1 ,C2 – capitate trichomes;
