# Supplementary material for: Essential Oil Composition and Micromorphological Traits of Satureja montana L., S. subspicata Bartel ex Vis., and S. kitaibelii Wierzb. Ex Heuff. Plant Organs
Source: Plants (Basel). 2021 Mar 9;10(3):511. doi: 10.3390/plants10030511 (PMC8000019; doi:10.3390/plants10030511)
Supplement: Supplementary file 1 [file Supplemental_S2.pdf › Supplemental_S2.pdf]

Table S2. Composition of plant and herba essential oil obtained from *Satureja montana, S. kitaibelii* and *S. Subspicata*

	Taxon		M1				M2				K				S1				S2			
#	**Compound**[1]	LRI[2]	Leaf	Calyx	Corolla	Herba	Leaf	Calyx	Corolla	Herba	Leaf	Calyx	Corolla	Herba	Leaf	Calyx	Corolla	Herba	Leaf	Calyx	Corolla	Herba
1	[2E]-Hexenal	867	-	-	-	-	-	-	-	-	-	-	-	-	-	-	-	-	0.3±0.3	-	-	-
2	Tricyclene	919	tr	-	-	-	-	-	-	-	-	-	-	-	0.2±0.1	-	1.0	-	-	-	-	-
3	α -Thujene	922	0.9±0.4	0.2	tr	0.4	1±0.2	1.8	0.7	1.0	0.1±0	-	-	0.2	0.3±0.1	0.6	0.6	0.2	-	-	-	-
4	α -Pinene	929	1.2±0.3	1.8	0.6	0.5	0.9±0.1	0.9	0.4	0.5	1.2±0	0.3	0.3	2.0	20.8±4.6	31.4	40.3	10.2	28.8±8.3	18.9	9.3	57.0
5	Camphene	944	1.1±0.4	0.3	0.1	0.4	0.4±0	0.3	0.1	0.3	0.5±0	-	0.5	0.9	6.6±1.4	7.4	10.2	3.0	0.1±0.1	0.2	-	0.6
6	Thuja-2,4(10)-diene	949	tr	-	-	-	-	-	-	-	-	-	-	-	tr	0.3	-	-	0.2±0.2	-	-	-
7	Sabinene	969	0.3±0.2	0.6	0.2	0.9	-	0.2	0.2	0.2	0.2±0.1	-	-	-	-	-	-	-	-	-	-	-
8	β -Pinene	973	0.3±0.3	0.6	-	0.3	0.2±0.4	0.6	tr	-	tr	-	-	0.4	1.2±0.3	1.2	1.2	0.6	0.6±0.2	0.3	-	0.9
9	1-Octen-3-ol	976	0.3±0.5	-	0.1	-	0.5±0.4	-	-	0.7	-	-	-	-	-	-	-	-	-	-	-	-
10	Myrcene	987	-	1.1	0.3	0.6	0.5±0.2	2.2	0.9	1.4	0.2±0.1	-	-	0.5	1.1±0.1	-	-	0.6	0.9±0.4	1.1	0.2	2.4
11	Dehydro-1,8-Cineole	988	0.2±0.1	-	-	-	-	-	-	-	-	-	-	-	-	-	-	-	-	-	-	-
12	3-Octanol	992	-	-	-	-	-	-	-	-	-	-	-	-	0.4±0	-	-	0.4	-	-	-	-
13	α -Phellandrene	1003	tr	0.2	-	0.1	tr	0.3	0.1	0.2	-	-	-	tr	-	-	-	-	-	0.1	-	0.2
14	α -Terpinene	1015	1.8±0.9	0.6	0.1	2.7	1.1±0.1	4.1	1.7	2.6	-	-	-	tr	0.2±0	0.3	-	0.2	-	-	-	-
15	*p* -Cymene	1027	33.5±5.6	6.8	1.3	13.4	30±4.3	3.9	4.3	11.9	13.1±2.2	-	-	13.2	3.1±0.5	2.0	1.4	2.2	0.3±0.1	0.5	-	0.8
16	Limonene	1028	1.5±0.2	2.8	1.0	1.2	0.8±0.2	0.6	0.6	0.6	3.1±0.1	0.5	0.5	4.5	7±1.1	6.3	4.0	4.9	1.2±0.5	1.5	0.4	3.3
17	1,8-Cineole	1030	0.7±0.2	0.2	-	0.1	0.3±0.2	0.2	-	0.2	0.9±0.1	-	-	0.8	4.5±0.5	7.1	6.1	4.7	-	-	-	-
18	*cis* -β -Ocimene	1037	-	1.3	0.4	0.1	-	-	-	-	0.4±0.1	-	-	0.9	-	-	-	-	-	0.1	-	0.4
19	*trans* -β -Ocimene	1047	-	0.4	-	-	-	0.1	-	0.1	0.3±0.1	-	-	1.7	-	-	-	-	-	-	-	-
20	γ -terpinene	1059	2.9±1.5	4.8	0.7	8.9	4.1±1.5	28.4	12.1	18.8	0.4±0	-	-	1.1	0.2±0	0.4	-	0.3	-	-	-	-
21	*cis* -Sabinene hydrate	1067	13.0±4.5	16.5	13.1	24.8	4.9±0.1	5.0	9.5	8.2	0.3±0.1	-	-	0.3	tr	2.3	1.2	1.0	-	-	-	-
22	*cis* -Linalool oxide (furanoid)	1072	0.4±0.8	-	0.3	-	-	-	-	-	1.7±0.3	-	-	0.8	-	-	-	-	-	-	-	-
23	*trans* -Linalool oxide (furanoid)	1090	0.5±0.9	-	0.3	-	0.6±0.1	0.3	0.4	0.6	1.7±0.3	-	-	0.8	-	-	-	-	-	tr	-	-
24	Terpinolene	1090	0.6±0.5	0.2	-	1.1	tr	0.1	-	0.1	-	-	-	-	tr	-	-	0.3	-	-	-	-
25	Linalool	1101	2.3±2.0	2.7	5.1	-	-	-	-	-	43.5±1	38.3	48.4	47.3	1.4±0.3	-	0.7	1.4	0.4±0.1	0.5	0.5	0.6
26	*trans* -Sabinene hydrate	1102	1.4±2.4	0.6	-	2.7	-	-	-	-	-	-	-	-	-	1.2	-	0.6	-	-	-	-
27	Isopentyl isovalerate	1109	-	-	-	-	-	-	-	-	-	-	-	-	-	-	-	-	0.2±0.2	-	-	0.2
28	α -Campholenal	1124	-	-	tr	-	-	-	-	-	-	-	-	-	tr	0.8	1.1	0.6	-	-	-	-
29	*cis* -*p* -Menth-2-en-1-ol	1123	0.7±0.1	0.2	0.2	1.0	-	-	-	-	-	-	-	-	-	-	-	-	-	-	-	-
30	*trans* -*p* -Mentha-2,8-dien-1-ol	1124	-	-	-	-	-	-	-	-	tr	-	-	tr	-	-	-	0.3	-	-	-	-
31	allo-Ocimene	1132	-	-	-	-	-	-	-	-	0±0.1	-	-	0.6	-	-	-	-	-	-	-	0.2
32	*trans* -*p* -Menth-2-en-1-ol	1140	0.5±0.0	-	0.1	0.5	-	-	-	-	-	-	-	-	-	-	-	-	-	-	-	-
33	*trans* -Pinocarveol	1140	-	-	-	-	-	-	-	-	0.1±0	-	-	tr	0.2±0	0.7	0.7	0.6	0.1±0.1	-	-	-
34	Camphor	1144	0.6±0.1	0.2	-	0.3	-	-	-	-	0.4±0.1	-	-	0.2	3.2±0.3	7.6	7.9	6.6	0.1±0.1	0.3	0.3	-
35	Sabina ketone	1157	tr	-	-	-	-	-	-	-	-	-	-	-	-	-	-	-	-	-	-	-
36	Borneol	1165	2.9±0.8	1.7	0.6	2.5	1.2±0.2	0.6	0.3	1.4	2.9±0.3	1.6	0.5	2.2	7.3±0.8	6.9	7.2	10.0	-	0.1	-	-
37	*cis* -Linalool oxide (pyranoid)	1169	0.1±0.1	-	-	-	-	-	-	-	0.4±0.1	-	-	0.2	-	-	-	-	-	-	-	-
38	*trans* -Linalool oxide (pyranoid)	1174	0.1±0.2	-	-	-	-	-	-	-	0.3±0.1	-	-	0.1	-	-	-	-	-	-	-	-
39	Terpinen-4-ol	1178	8.9±1.0	2.1	2.3	13.0	0.7±0.1	0.5	0.6	0.7	0.2±0	-	-	0.1	4±0.6	3.0	1.6	5.5	-	tr	-	-
40	*p* -Cymen-8-ol	1185	1.9±0.3	0.4	0.4	0.9	0.2±0	-	-	tr	0.7±0.2	-	-	0.3	0.2±0.1	-	-	0.6	-	-	-	-
41	α -Terpineol	1190	1.7±0.8	1.5	1.4	5.4	0.5±0.1	0.5	0.4	0.7	0.2±0	-	-	0.1	1.4±0.5	0.8	-	2.1	-	0.1	-	-
42	*cis* -Piperitol	1194	0.4±0.1	-	0.2	0.2	-	-	-	-	-	-	-	-	-	-	-	-	-	-	-	-
43	*cis* -Dihydro carvone	1196	0.3±0.0	-	-	-	-	-	-	-	0.4±0	-	-	0.2	-	-	-	-	-	-	-	-
44	*trans* -Dihydro carvone	1203	-	-	-	-	-	-	-	-	0.9±0.3	0.2	-	0.8	-	-	-	-	-	-	-	-
45	Verbenone	1208	-	-	0.1	-	-	-	-	-	0.1±0	-	-	-	tr	1.2	1.4	1.1	-	0.2	-	-
46	*trans* -Carveol	1218	0.1±0.1	-	0.2	-	-	-	-	-	0.3±0.1	-	-	0.1	tr	-	-	0.7	-	-	-	-
47	Nerol	1228	0.2±0.4	2.5	1.4	-	-	-	-	-	-	-	-	-	-	-	-	-	-	-	-	-
48	(3*Z*)-Hexenyl 2-methyl butanoate	1231	0.1±0.1	-	-	-	-	-	-	-	-	-	-	-	-	-	-	-	tr	-	-	0.4
49	Thymol methyl ether	1233	-	-	-	-	0.1±0.1	0.3	0.2	tr	-	-	-	-	-	-	-	-	-	-	-	-
50	(3*Z*)-Hexenyl 3-methyl butanoate	1236	-	-	-	-	-	-	-	-	-	-	-	-	-	-	-	-	0.2±0.2	0.7	-	-
51	Cumin aldehyde	1239	0.2±0.2	-	-	0.2	-	-	-	-	tr	-	-	-	-	-	-	-	-	-	-	-
52	Neral	1240	0.4±0.7	1.2	2.0	-	-	-	-	-	-	-	-	-	-	-	-	-	-	-	-	-
53	Carvacrol methyl ether	1243	2.1±3.1	-	-	-	-	-	-	-	-	-	-	-	-	-	-	-	-	-	-	-
54	Carvone	1244	-	-	0.2	0.1	-	-	-	-	0.3±0	-	-	0.1	-	-	-	-	-	-	-	-
55	Thymoquinone	1249	0.1±0.1	-	-	-	11±2.5	0.2	1.5	1.6	0.6±0.1	-	-	0.2	-	-	-	-	-	-	-	-
56	Geraniol	1257	0.8±1.3	19.0	30.4	-	-	-	-	-	-	-	-	-	-	-	-	-	-	-	-	-
57	Geranial	1270	0.4±0.7	2.4	4.7	-	-	-	-	-	-	-	-	-	-	-	-	-	-	-	-	-
58	Bornyl acetate	1285	0.4±0.3	0.2	0.1	0.4	-	-	-	-	-	-	-	-	3±0.8	0.5	1.3	3.1	0.2±0	0.5	0.3	0.2
59	*p* -Cymen-7-ol	1291	0.3±0.1	-	-	0.2	tr	-	-	-	-	-	-	-	-	-	-	-	-	-	-	-
60	Thymol	1292	0.4±0.2	0.2	0.1	0.3	29.6±3.4	39.4	51.7	40.5	1±0.2	-	-	0.5	-	-	-	-	-	-	-	-
61	Carvacrol	1302	1.2±0.5	-	-	0.8	4.6±0.6	4.4	6.3	4.8	1.4±0.2	-	-	0.9	-	-	-	-	-	-	-	-
62	Geranyl formate	1302	-	0.2	0.3	-	tr	-	-	-	-	-	-	-	-	-	-	-	-	-	-	-
63	δ -Elemene	1336	-	-	-	-	-	-	-	-	0.2±0	5.9	0.4	0.9	-	-	-	-	1.1±0.1	4.3	0.5	1.4
64	α -Cubebene	1350	-	-	-	-	-	-	-	-	-	-	-	-	-	-	-	-	0.7±0.1	tr	-	0.3
65	α -Ylangene	1371	-	-	-	-	-	-	-	-	-	-	-	-	tr	-	-	-	0.2±0.2	-	-	-
66	α -Copaene	1375	tr	-	0.2	tr	-	-	-	-	0.3±0	0.3	0.3	0.2	0.3±0	-	-	0.3	1.5±0.1	0.7	1.2	0.8
67	β -Bourbonene	1384	0.8±1.1	-	5.9	0.2	-	-	-	-	1±0.2	0.5	0.4	0.6	0.8±0.1	-	-	0.8	4.5±0.2	1.5	1.2	2.0
68	β -Elemene	1391	-	0.4	0.3	0.2	-	-	-	-	0.3±0	2.5	1.8	0.5	1.6±0.2	3.2	2.5	3.1	1.3±0.1	5.3	5.5	1.7
69	α -Gurjunene	1409	-	-	-	-	-	-	-	-	tr	0.2	-	tr	1.4±0.2	0.8	0.3	1.6	0.4±0	0.3	-	0.3
70	(*E*)-Caryophyllene	1418	0.4±0.2	6.3	4.4	2.6	2.2±0.4	2.9	5.0	1.0	2.4±0.2	17.1	21.3	3.8	1.4±0.3	0.4	0.4	1.5	3.8±0.2	5.5	8.4	2.9
71	β -Copaene	1428	tr	-	0.1	tr	-	tr	tr	-	0.4±0.1	3.5	3.3	0.8	0.2±0	-	-	0.2	2.2±0.1	2.3	3.2	1.4
72	*cis* -Muurola-3,5-diene	1444	-	-	-	-	-	-	-	-	-	-	-	-	-	-	-	-	0.6±0	0.9	1.2	0.5
73	Aromadendrene	1445	tr	-	0.1	tr	-	-	-	-	-	-	-	-	-	-	-	-	0.5±0	0.2	-	-
74	*trans* -Muurola-3,5-diene	1453	-	-	-	-	-	-	-	-	-	-	-	-	-	-	-	-	0.3±0.3	1.1	1.4	0.5
75	α -Humulene	1455	tr	0.4	0.4	0.1	-	tr	0.2	tr	0.1±0.1	-	-	-	0.1±0.1	-	-	0.2	0.2±0.3	-	-	-
76	allo-Aromadendrene	1460	tr	-	-	-	-	-	-	-	-	-	-	-	-	-	-	-	0.2±0.2	0.2	0.1	-
77	*cis* -Cadina-1(6),4-diene	1460	-	-	-	-	-	-	-	-	tr	0.6	0.6	tr	-	-	-	-	0.5±0.1	-	-	-
78	9-*epi* -(*E*)-Caryophyllene	1462	-	-	-	-	-	-	-	-	-	-	-	-	0.2±0	-	-	0.2	-	-	-	-
79	*cis* -Muurola-4(14),5-diene	1466	-	-	-	-	-	-	-	-	-	-	-	-	-	-	-	-	0.3±0.1	0.4	0.4	0.2
80	4,5-*di-epi* -Aristolochene	1468	-	-	-	-	-	-	-	-	-	-	-	-	0.1±0	-	-	0.1	-	-	-	-
81	γ-Muurolene	1479	-	-	tr	-	-	tr	tr	tr	0.2±0.1	0.5	-	0.1	1±0.2	-	-	1.0	3±0.4	1.0	1.1	1.1
82	Germacrene D	1483	tr	6.0	5.6	3.1	-	0.4	0.9	0.2	2.3±1.1	16.0	15.9	3.0	-	-	-	-	4.9±0.2	16.5	26.2	5.8
83	β -Selinene	1488	-	-	-	-	-	-	-	-	-	-	-	-	4.1±0.6	1.3	1.2	4.1	1.8±0.3	0.7	1.1	0.7
84	α -Selinene	1495	0.1±0.3	-	-	-	-	-	-	-	-	-	-	-	3.5±0.5	0.7	0.7	3.3	0.9±1.5	-	-	-
85	*cis* -β -Guaiene	1495	-	-	-	-	-	-	-	-	-	-	-	-	-	-	-	-	1.4±1.3	-	-	-
86	Bicyclogermacrene	1496	tr	1.1	0.4	2.8	-	0.2	-	tr	0.6±0.3	4.8	1.3	0.9	-	-	-	-	1.8±0.3	7.6	0.4	2.6
87	α -Muurolene	1500	-	-	tr	-	-	-	-	-	tr	-	-	-	0.3±0	-	-	0.3	0.5±0	0.2	1.7	-
88	β -Bisabolene	1511	0.3±0.1	1.4	0.2	0.8	0.5±0.5	0.7	0.5	0.2	0.8±0	-	-	0.3	-	-	-	-	-	0.3	-	-
89	γ -Cadinene	1514	tr	-	0.2	tr	-	0.2	0.5	tr	0.2±0.1	-	-	tr	0.8±0.1	0.4	0.6	0.9	1.6±0.2	0.5	0.6	0.5
90	7-*epi* -α -Selinene	1519	-	-	-	-	-	-	-	-	-	-	-	-	0.2±0	-	-	-	-	-	-	-
91	δ -Cadinene	1523	-	0.4	0.5	0.2	tr	0.1	0.2	tr	0.2±0.1	0.5	-	0.1	1.2±0.1	0.3	0.4	1.0	2.9±0.5	0.9	1.1	1.0
92	Dihydroactinidiolide	1528	-	-	-	-	-	-	-	-	-	-	-	-	tr	-	-	-	tr	-	-	-
93	α-Cadinene	1540	-	-	-	-	-	-	-	-	-	-	-	-	0.1±0	-	-	-	0.2±0	-	-	-
94	Unknown 1	1553	-	-	-	-	-	-	-	-	0.1±0.1	-	-	-	tr	-	-	-	-	0.1	0.5	-
95	Elemol	1550	0.1±0.1	0.9	0.4	1.4	-	-	-	-	-	-	-	-	-	-	-	-	-	-	-	-
96	Germacrene D-4-ol	1576	-	1.2	1.5	0.2	-	-	-	-	-	-	-	-	-	-	-	-	-	-	-	-
97	Spathulenol	1578	2.5±0.3	0.7	0.4	1.4	tr	-	-	tr	2.2±0.2	1.4	-	1.0	1.1±0.2	1.5	-	1.6	10.9±2.8	8.7	3.7	4.5
98	Caryophyllene oxide	1583	3.0±0.8	2.4	3.4	1.2	1.7±0.2	tr	0.1	0.4	3.4±0.2	0.6	-	1.4	1.7±0.2	3.4	3.1	2.9	1.9±0.5	0.9	1.1	0.7
99	β -Copaene-4-α -ol	1587	-	-	-	-	-	-	-	-	-	-	-	-	tr	-	-	-	0.5±0.1	-	-	-
100	Salvial-4(14)-en-1-one	1591	-	-	-	-	-	-	-	-	tr	-	-	-	0.3±0.1	-	0.3	0.4	0.8±0.2	0.6	1.2	0.2
101	Unknown 2	1602	-	-	-	-	-	-	-	-	-	-	-	-	tr	-	-	0.1	0.2±0.2	-	-	-
102	Unknown 3	1608	0.1±0.1	-	0.3	-	-	-	-	-	0.1±0	-	-	-	-	-	-	-	0.7±0.2	-	-	-
103	β -Oplopenone	1610	0.1±0.2	-	0.3	-	-	-	-	-	0.3±0.1	-	-	0.1	0.5±0.1	-	0.3	0.5	0.5±0.2	-	-	-
104	1,10-di-*epi* -Cubenol	1614	-	-	0.3	-	-	-	-	-	-	-	-	-	0.5±0.1	-	-	0.5	-	-	-	-
105	1-*epi* -Cubenol	1630	-	-	-	-	-	-	-	-	-	-	-	-	0.4±0.1	-	-	0.3	0.2±0.2	-	-	-
106	Caryophylla-3(15),7(14)-dien-6-α -ol	1634	-	-	-	tr	-	-	-	-	-	-	-	-	tr	-	-	0.1	1.1±0.3	-	-	-
107	Caryophylla-4(12),8(13)-dien-5-β -ol	1638	0.4±0.1	-	-	-	-	-	-	-	0.3±0	-	-	tr	0.4±0.2	-	-	0.5	-	0.7	0.3	-
108	*epi* -α -Cadinol	1641	-	-	-	-	-	-	-	-	-	-	-	-	0.7±0.6	-	-	-	1.2±0.4	0.5	0.2	-
109	α -Muurolol	1645	-	-	0.3	-	-	-	-	-	-	0.4	-	-	0.7±0.6	-	0.3	0.9	0.6±0.3	-	-	-
110	Unknown 4	1648	0.1±0.0	-	-	-	-	-	-	-	0.2±0.2	-	-	-	-	-	-	-	-	0.2	0.3	0.3
111	Cubenol	1649	-	-	-	-	-	-	-	-	-	-	-	-	-	-	-	-	0.4±0.2	0.3	0.6	-
112	β -Eudesmol	1653	-	-	-	-	-	-	-	-	-	-	-	-	2±1.6	-	-	-	-	-	-	-
113	Unknown 5	1651	-	-	-	-	-	-	-	-	tr	-	-	-	tr	-	-	-	tr	-	-	-
114	Unknown 6	1655	-	-	-	-	-	-	-	-	0.2±0.2	-	-	-	-	-	-	-	0.5±0.3	-	0.3	0.2
115	α -Cadinol	1657	0.1±0.1	0.5	0.7	0.2	-	-	-	-	-	-	-	-	1.3±2.2	-	-	2.9	3.3±1.1	1.4	2.4	0.9
116	Selin-11-en-4-α -ol	1656	0.3±0.2	-	-	-	-	-	-	-	-	-	-	-	1.1±0.3	0.4	0.6	1.2	-	-	-	-
117	Unknown 7	1658	-	-	-	-	-	-	-	-	0.2±0.2	-	-	-	-	-	-	-	-	-	0.5	-
118	14-Hydroxy-β -caryophyllene	1673	0.5±0.1	0.2	0.4	0.3	0.1±0.1	-	-	-	0.4±0.1	-	-	0.1	0.6±0.3	0.8	0.7	1.1	-	-	-	-
119	Eudesma-4(15),7-dien-1-β -ol	1685	-	-	-	-	-	-	-	-	0.6±0.1	-	-	0.3	-	-	-	-	3.2±1.3	1.6	2.8	1.2
120	Germacra-4(15),5,10(14)-trien-1-α -ol	1688	-	-	0.3	-	-	-	-	-	-	-	-	-	0.8±0.3	-	-	0.8	-	-	-	-
	**Total monoterpenes**		**87.4±4.4**	**73.7**	**68.3**	**84.0**	**92.9±2.4**	**95.1**	**91.9**	**96.4**	**78.2±1.8**	**41.0**	**50.1**	**82.0**	**66.3±6.6**	**82.2**	**88.0**	**61.2**	**33.2±10.2**	**24.4**	**11.0**	**66.8**
	Monoterpene hydrocarbons		*44.3±8.2*	*21.8*	*4.7*	*30.6*	*39±3.5*	*43.5*	*21.1*	*37.7*	*19.7±2.6*	*0.9*	*1.3*	*25.9*	*40.8±8.3*	*49.9*	*58.8*	*22.4*	*32.1±9.7*	*22.7*	*9.9*	*65.8*
	Oxygenated monoterpenes		*43.1±5.0*	*51.9*	*63.6*	*53.3*	*53.9±2.6*	*51.5*	*70.9*	*58.7*	*58.5±1*	*40.1*	*48.8*	*56.1*	*25.5±3.3*	*32.3*	*29.3*	*38.9*	*1.1±0.6*	*1.8*	*1.1*	*1.0*
	**Total sesquiterepenes**		**8.9±2.4**	**21.9**	**26.2**	**14.7**	**4.6±1.2**	**4.5**	**7.5**	**1.8**	**16.4±1.6**	**54.9**	**45.3**	**14.3**	**29.8±5.4**	**13.3**	**11.3**	**32.4**	**62±9.4**	**64.9**	**67.4**	**31.2**
	Sesquiterepen hydrocarbons		*2.1±1.3*	*15.9*	*18.3*	*10.0*	*2.7±0.9*	*4.5*	*7.4*	*1.4*	*9.1±1.7*	*52.4*	*45.3*	*11.3*	*17.4±2.4*	*7.2*	*6.0*	*18.7*	*37.4±1.8*	*50.3*	*55.3*	*23.6*
	Oxygenated sesquiterpenes		*6.8±1.3*	*6.0*	*7.9*	*4.8*	*1.9±0.3*	*0.0*	*0.1*	*0.4*	*7.3±0.6*	*2.4*	*0.0*	*3.0*	*12.4±3.1*	*6.1*	*5.3*	*13.7*	*24.6±7.5*	*14.6*	*12.1*	*7.6*
	**Other**[3]		**0.5±0.6**	**0.0**	**0.1**	**0.0**	**0.5±0.4**	**0.0**	**0.0**	**0.7**	**0±0**	**0.0**	**0.0**	**0.0**	**0.4±0**	**0.0**	**0.0**	**0.4**	**0.6±0.4**	**0.7**	**0.0**	**0.4**
	**Unkown**		**0.1±0.1**	**0.0**	**0.3**	**0.0**	**0±0**	**0.0**	**0.0**	**0.0**	**0.9±0.7**	**0.0**	**0.0**	**0.0**	**0.2±0.3**	**0.0**	**0.0**	**0.1**	**1.5±0.5**	**0.3**	**1.7**	**0.4**
	**No of compounds**		**52-56**	**44**	**54**	**44**	**23-28**	**29**	**26**	**26**	**57-60**	**20**	**14**	**46**	**56-59**	**31**	**30**	**55**	**46-52**	**45**	**35**	**37**
	**Total**		**97.0±1.6**	**95.6**	**94.9**	**98.7**	**98.1±1**	**99.6**	**99.4**	**99.0**	**95.5±0.6**	**95.9**	**95.4**	**96.3**	**96.8±1.3**	**95.5**	**99.4**	**94.1**	**97.3±0.9**	**90.3**	**80.1**	**98.8**

[1], Contents of the total essential oil composition are given as percentages, in leaf essential oil as mean ± SD; tr: trace (0.05 < tr < 0.10%); –: not detected; compounds with contents <0.05% are not shown, [2] Experimental LRIs were determined using the standard index method involving retention time (tR) of *n* -alkanes injected under the same chromatographic conditions; [3] Other: diterpenes and non-terpenoid compounds, i.e. aliphatic hydrocarbons, aliphatic aldehydes and alcohols, aliphatic acids, their esters and aldehydes, aromatic esters with aliphatic acids, alkyl- aromatic alcohols, and aryl esters of aromatic acids. M1 – *Satureja montana* from mountain region; M2 – *S. montana* from coastal region; K – *S. kitaibelii* ; S1 – *S. subspicata* subps. *liburnica* ; S2 – *S. subspicata* subsp. *subspicata* ; P – peltate trichomes; C1 ,C2 – capitate trichomes;
